# Stability of neuronal avalanches and long-range temporal correlations during the first year of life in human infants

**DOI:** 10.1007/s00429-019-02014-4

**Published:** 2020-02-24

**Authors:** Mostafa Jannesari, Alireza Saeedi, Marzieh Zare, Silvia Ortiz-Mantilla, Dietmar Plenz, April A. Benasich

**Affiliations:** 1 School of Computer Science, Institute for Research in Fundamental Sciences (IPM), 70 Lavasani Avenue, Tehran, 19395 Iran; 2 Department of Physiology of Cognitive Processes, Max-Planck-Institute for Biological Cybernetics, 72076 Tübingen, Germany; 3 Center for Molecular and Behavioral Neuroscience, Rutgers University-Newark, 197 University Avenue, Newark, NJ 07102 USA; 4 Section on Critical Brain Dynamics, Laboratory of Systems Neuroscience, National Institute of Mental Health, Porter Neuroscience Research Center, MSC 3735, Bethesda, MD 20892 USA

**Keywords:** Neuronal avalanches, Infancy, Criticality, EEG

## Abstract

During infancy, the human brain rapidly expands in size and complexity as neural networks mature and new information is incorporated at an accelerating pace. Recently, it was shown that single-electrode EEG in preterms at birth exhibits scale-invariant intermittent bursts. Yet, it is currently not known whether the normal infant brain, in particular, the cortex, maintains a distinct dynamical state during development that is characterized by scale-invariant spatial as well as temporal aspects. Here we employ dense-array EEG recordings acquired from the same infants at 6 and 12 months of age to characterize brain activity during an auditory odd-ball task. We show that suprathreshold events organize as spatiotemporal clusters whose size and duration are power-law distributed, the hallmark of neuronal avalanches. Time series of local suprathreshold EEG events display significant long-range temporal correlations (LRTCs). No differences were found between 6 and 12 months, demonstrating stability of avalanche dynamics and LRTCs during the first year after birth. These findings demonstrate that the infant brain is characterized by distinct spatiotemporal dynamical aspects that are in line with expectations of a critical cortical state. We suggest that critical state dynamics, which theory and experiments have shown to be beneficial for numerous aspects of information processing, are maintained by the infant brain to process an increasingly complex environment during development.

## Introduction

This article replaces an incorrect version that was previously published in error [Jannesari, M., Saeedi, A., Zare, M., Ortiz-Mantilla, S., Plenz, D., & Benasich, A. A. (2019). Stability of neuronal avalanches and long-range temporal correlations during the first year of life in human infant. Brain Structure and Function, 224(7), 2453–2465. 10.1007/s00429-019-01918-5. Epub 2019 Jul 2.].

Studies on early human development, in both preterm and term newborns, have revealed structural aspects of early human brain growth using magnetic resonance imaging (MRI) (Kostović and Judaš 2006, 2008; Hüppi et al. 1998), diffusion tensor imaging (DTI) (Hüppi and Dubois 2006), and histological preparations (Kostović and Judaš 2006, 2008). Converging quantitative EEG and volumetric MRI analyses demonstrating significant correlation between early higher brain activity levels and increased brain volumes in preterm newborns (Benders et al. 2015) support the idea that neuronal growth and survival are sensitive to early network activity.

Whereas the mentioned studies have focused on early developmental changes in brain growth and brain activity from a broader point of view, recent studies have taken these investigations a step further by examining stability in neuronal network function, essentially, the role of brain activity in homeostasis (Prinz et al. 2004; Bucher et al. 2005; Marder and Goaillard 2006). Homeostasis and dynamical regulation of brain networks require detection of scale-invariant neuronal bursts of activity. The most common scale-invariant dynamics in cortex are neuronal avalanches (Beggs and Plenz 2003) and to date they have been demonstrated in adult humans in EEG (Meisel et al. 2013; Allegrini et al. 2010; Benayoun et al. 2010), ECoG (Solovey et al. 2012; Priesemann et al. 2013), MEG (Palva et al. 2013; Shriki et al. 2013), and fMRI (Tagliazucchi et al. 2012).

Rapidly accumulating evidence supports the hypothesis that cortical dynamics in humans exhibit scale-invariant features that change with altered states such as sleep deprivation (Meisel et al. 2013, 2017a, b) and epilepsy (Arviv et al. 2016). In infants, scale-invariant neuronal activity has been observed in the single-electrode EEG of preterm infants as early as 12 h after birth (Iyer et al. 2015). Importantly, deviations from scale-invariant neuronal activity have been shown to track recovery from the burst suppression induced by hypoxia (Roberts et al. 2014) and to predict recovery from early hypoxic insult (Iyer et al. 2014). This suggests that measures of scale-invariant dynamics may serve as potential biomarkers for early detection of concurrent risk and/or disease in infants. Nonetheless, due to the fact that the scale-invariant activity present at those early ages has been pathological in nature, to date only associated with clinical conditions (i.e. prematurity or hypoxia at birth), it is unclear whether this neural activity is categorically the same in nature as neuronal avalanches described in adults.

Furthermore, the time course and the evolving characteristics of scale-invariant cortical dynamics over normative development are also unknown. In addition, it is uncertain to what degree brain dynamics are stable over the first year of life when the infant brain dramatically expands in size and changes in connectivity, a challenge posited by Marder et al. as well as other researchers (Prinz et al. 2004; Bucher et al. 2005; Marder and Goaillard 2006). As noted, neuronal avalanche-like activity has been identified in infant clinical populations using a single-electrode EEG sensor in a narrow frequency band (Roberts et al. 2014; Iyer et al. 2014, 2015). But to examine avalanche dynamics across typical development with a more holistic perspective, broadband multi-site recordings of cortical activity that allow global and local analyses would be more appropriate (Grieve et al. 2003; Odabaee et al. 2013).

The focus of the present study, in which dense-array EEG data were recorded while infants listened to tone-pairs presented in a passive oddball paradigm, was twofold: (1) to explore ongoing neocortical activities for potential neuronal avalanche dynamics, and (2) to identify the degree of stability of avalanche dynamics over the course of 6 months of normative development.

## Materials and methods

### Participants

Participants in the current study were a subset of children who had participated in a larger longitudinal study that assessed the effects of early auditory processing skills on later language and cognitive development (Benasich et al. 2006; Choudhury et al. 2007; Choudhury and Benasich 2011; Benasich and Choudhury 2012). Infants were recruited from local newspapers, birth announcements, and pediatric clinics. The infant group consisted of 19 typically developing full-term, normal birthweight infants (11 males, 8 females) with no reported family history of developmental language disorders (DLD). They were tested longitudinally at each of seven ages from 6 months through 48 months of age using both behavioral and electrophysiological assessments. For the purpose of the present study, only the 6- and 12-month EEG recordings were used. This study was approved by the Institutional Review Board of Rutgers University, and conducted in accordance with the 1964 declaration of Helsinki. Informed consent was obtained from all parents following a full explanation of the experiment and prior to their child inclusion in the study.

### EEG stimuli and recording

The EEG was recorded following our infant protocol (Musacchia et al. 2015) while the infants were awake and comfortably seated on their parent’s lap in a sound-attenuated and electrically shielded chamber. Silent videos were played on a monitor to engage the infant’s attention and minimize movements. If the infant lost interest in the video, an experimenter played a silent puppet show or used quiet toys. EEG was recorded from 62 scalp sites using the Geodesic Sensor Net (Electrical Geodesics Inc., Eugene, OR). The signal was sampled at 250 Hz, referenced on-line to the vertex and band-pass filtered at 0.1–100 Hz. For the avalanche analysis, EEG recordings were re-referenced to an average (whole head) reference. The auditory stimuli used were complex tone-pairs, each tone 70 ms in duration. The first block presented was a fast-rate block with an interstimulus interval (ISI) of 70ms. The second block was a slow-rate block with 300 ms ISI. Between the blocks, a short period of 3–4 min of ongoing spontaneous activity without auditory stimulation was recorded. Tone-pairs were presented in a passive oddball paradigm with the standard pair (100–100 Hz) presented 85% of the time and the deviant pair (100–300 Hz) randomly presented 15% of the time. Preliminary examination of the spontaneous block showed power-law behavior but included high fluctuations in the avalanche profile that lowered the level of confidence required for this type of analysis. However, note that it has been demonstrated that evoked responses do not affect power-law statistics and that evoked data preserve the signature of neuronal avalanches (Yu et al. 2017). Thus, for the purpose of this study, only results from the fast-rate (70 ms ISI) condition are reported because (1) the duration of that block allowed the continuous data points necessary to conduct the avalanche analysis and, as mentioned, (2) given the high fluctuation in avalanche profile observed in the spontaneous block, our confidence in the stability and reliability of that analysis was low. The average duration of the EEG recording was 12 min for each child for the 70 ms ISI condition. For a more detailed description of the stimuli and paradigm used, please refer to Benasich et al. (2006). Several noisy channels were identified for a subset of the subjects; they were removed and interpolated via automatic channel rejection (EEGLAB) (Delorme and Makeig 2004) using the Kurtosis measure. In particular, two channels at 6 months and three channels at 12 months were interpolated for the subject reported here (subject #14). As suggested by Odabaee et al. (2013), due to the spatial specificity of infant data, a high number of EEG channels is required to have a precise and reliable analysis. Here, however, we have 62 channels and the average ratio of interpolated channels is very low ($\sim 3\%$). Time series were broken into six frequency bands: delta ($\delta$, 0.5–4 Hz), theta ($\theta$, 4–8 Hz), alpha ($\alpha$, 8–13 Hz), beta ($\beta$, 13–30 Hz), low gamma ($\gamma_L$, 30–48 Hz), and high gamma ($\gamma _H$, 48–70 Hz). In addition to the six frequency bands, the full frequency band was also examined. MATLAB Signal Processing Toolbox and in-house functions were used for filtering the data (R2016; The Mathworks, Natick, MA).

### Avalanche detection

**Fig. 1:**
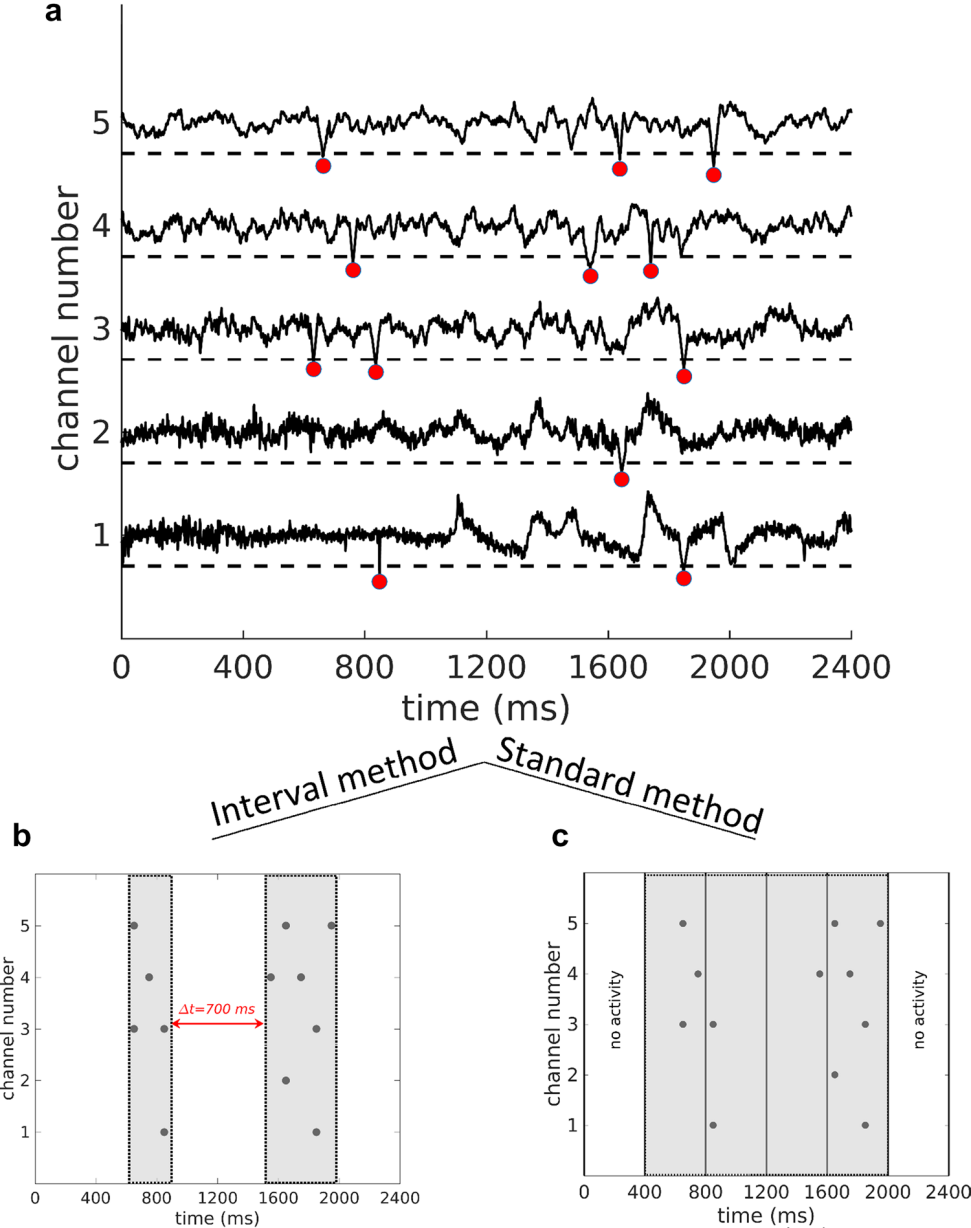
Brain activity and definition of avalanches. **a** EEG time course of five electrodes. Peak time and peak amplitude are extracted from suprathreshold negative (or positive) EEG deflections that cross a constant threshold set at a multiple of the EEG SD (all here were negative). Schematic highlighting differences in avalanche identification using **b** the interval method and **c** the standard method. The standard method identifies one avalanche based on four consecutive active bins, whereas the interval method identifies two avalanches based on two events exhibiting a time interval > 400 ms

Neuronal avalanches are defined as spatiotemporal clusters of neuronal activity within intervals less than a specific time regardless of electrode location (Beggs and Plenz 2003; Gireesh and Plenz 2008; Benayoun et al. 2010; Dehghani et al. 2012). Super-thresholded negative (or positive) peaks are defined as events in our analysis, and we performed four steps to identify events, and define neuronal cluster to analyze them in the infant EEG:

1. *z*-Transform the EEG time series at each electrode, i.e. subtract the mean and divide by the standard deviation (SD).
2. Set successive thresholds as multiples of SD ranging from 2 to 4 in steps of 0.25 and identify threshold crossings as shown in Fig. 1a. Mark peak time and peak amplitude of the *z*-normalized suprathreshold EEG potential for cluster calculations.
3. Define a separation time, $\Delta t$, between two consecutive suprathreshold events. Analyze neuronal clusters for a range of $\Delta t$.
4. Identify a neuronal cluster as consecutive events composed of all channels, in which the time interval between peaks across changes was smaller than $\Delta t$ (Fig. 1). This method has been previously used by Benayoun et al. (2010). In an identified neuronal cluster, avalanche size is defined as the number of super-thresholded negative (or positive) peaks (i.e. events) and avalanche duration is defined as the time distance between the first and last events in that cluster (note that size and duration are dimensionless).

Our method identifies spatiotemporal avalanches as consecutive periods of activity separated by more than $\Delta t$ (as illustrated in Fig. 1). This method differs from the avalanche algorithm (standard method), originally introduced by Beggs and Plenz (2003), which considers an avalanche as a sequence of time bins $\Delta t$ with activity bracketed by at least two time bins without activity (Fig. 1b). As illustrated in Fig. 1, the two methods lead to a slightly different partitioning of activity into temporal clusters. Using the interval method allowed us to easily make an average over all inter-event intervals, and select an appropriate time span to choose $\Delta t$. Moreover, this method provides more precise statistics on the timescales, leading to higher resolution/more accurate plots of the data. Due to the noisy nature of EEG data, the results from the two methods are similar, but not completely overlapping. Nonetheless, we found no significant difference ($P>0.05$) between the exponents derived from each method. Thus, we decided, in the interest of clarity and readability, to only report the results from the interval method.

It was previously shown that power-law behavior in avalanche sizes does not depend on the choice of separation time for a wide range of $\Delta t$s. In fact, scaling analysis for $\Delta t$ has been demonstrated to collapse avalanche power-laws (Beggs and Plenz 2003; Petermann et al. 2009; Yang et al. 2012). Similarly, power-law behavior in avalanche dynamics has been demonstrated to be largely threshold independent (Petermann et al. 2009; Tagliazucchi et al. 2012).

### Size and duration distributions of avalanches

It has been shown that avalanche distributions in size, *s*, and duration, *t*, exhibit a power-law behavior (Beggs and Plenz 2003; Shew et al. 2009; Friedman et al. 2012):

1 $$ \left\{ {\begin{array}{*{20}l}    {p(s) \propto s^{{ - \tau }} } \hfill  \\    {p(T) \propto T^{{ - \alpha }} } \hfill  \\   \end{array} ,} \right. $$

where *p* is the probability density function of the associated variables. To study the type of function underlying the avalanche distributions in size and duration obtained from the infant EEG, we used the maximum likelihood estimation (MLE) defined for an arbitrary distribution function (Clauset et al. 2009) as

2 $$ L(\alpha ,\beta , \ldots ) = \prod\limits_{{i = 1}}^{N} f (x_{i} ,\alpha ,\beta , \ldots ), $$

where $f(x_i)$ is the probability of *i*th data point. The $\alpha ,\beta , \ldots$ are the hypothetical model’s parameters and if chosen correctly, the likelihood will be maximized. Hence, we can choose any hypothetical model for distribution and find the model’s parameters by maximizing the likelihood. The maximization of the likelihood can be accomplished by maximizing its logarithm. Therefore, if the distribution is a power-law, we can calculate the logarithm of the likelihood for different exponents and find the corresponding exponent for the maximum likelihood. The normalized equation for discrete power-law distribution with low and high cutoff is

3 $$ f(x) = A(\alpha ,x_{{\min }} ,x_{{\max }} )\left( {\frac{1}{x}} \right)^{\alpha } , $$

where $\alpha$ is exponent and *A* is normalization factor:

4 $$ A(\alpha ,x_{{\min }} ,x_{{\max }} ) = \frac{1}{{\sum\limits_{{x = x_{{\min }} }}^{{x_{{\max }} }} {\left( {\frac{1}{x}} \right)^{\alpha } } }}. $$

Using Eqs. (2) and  (3), the logarithm of likelihood for power-law distribution is

5 $$ l(\alpha ) \equiv \frac{{\log (L(\alpha ))}}{N} =  - \log \left( {\sum\limits_{{x = x_{{\min }} }}^{{x_{{\max }} }} {\left( {\frac{1}{x}} \right)^{\alpha } } } \right) - \frac{\alpha }{N}\sum\limits_{{i = 1}}^{N} {\log (x_{i} )} , $$

where *N* is the number of data point between cutoffs.

**Fig. 2:**
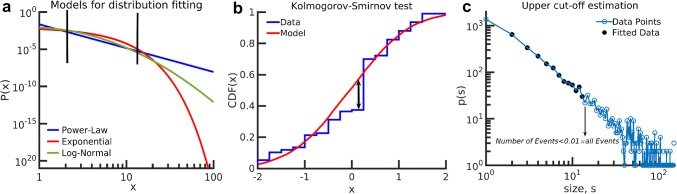
**a** Typical distribution of three models in a bi-logarithmic scale. Distributions show a line in a specific range of *x* while the exponential and log-normal models decrease faster in the tail. **b** The Kolmogorov–Smirnov test computed between the typical model and data cumulative distribution. The maximum distance between the cumulative distribution function is used as the criteria for the difference between distributions. **c** The estimation method of fitting range. The low cutoff is set to 2 and the high cutoff is determined by the probability of the avalanches. The high cutoff is the largest occurrence with $p(x) > 0.01$

The selection of low and high cutoffs, i.e. $x_{{\min }}$ and $x_{{\max }}$ affects the model estimation, and we will introduce a rational method for choosing them. Nevertheless, there are other distributions which show a semi-line on bi-logarithmic axes, such as exponential distribution and log-normal distribution (see Fig. 2a). We can also fit these distributions by the MLE method and find the model parameters. The probability density function (PDF) of an exponential distribution is

6 $$f(x) = \lambda e^{{ - \lambda x}} ,\quad x \ge 0,$$

where $\lambda$ is the model’s parameter. Using Eq. (2), the logarithm of likelihood for the exponential distribution is

7 $$ l(\lambda ) = \log (\lambda ) - \lambda \sum\limits_{{i = 1}}^{N} {\frac{{x_{i} }}{N}} . $$

For log-normal distribution, the probability density function is

8 $$ f(x) = \frac{1}{{\sqrt {2\pi } \sigma x}}{\text{e}}^{{ - \frac{{(\log (x) - \mu )^{2} }}{{2\sigma ^{2} }}}} , $$

where $\sigma$ and $\mu$ are model’s parameters. The logarithm of likelihood is

9 $$ \begin{aligned}   l(\sigma ,\mu ) &  =  - \frac{{\log (2\pi )}}{2} - \log (\sigma ) - \sum\limits_{{i = 1}}^{N} {\frac{{\log (x_{i} )}}{N}}  \\    & \quad  - \sum\limits_{{i = 1}}^{N} {\frac{{(\log (x_{i} ) - \mu )^{2} }}{{2N\sigma ^{2} }}} . \\  \end{aligned}  $$

The MLE method does not provide a quantitative value describing the accuracy of the hypothetical model. Therefore, a method of comparing different models was needed. By calculating a *P* value for each model, we can reject the inappropriate models. Here, we used the Kolmogorov–Smirnov (KS) test to calculate the difference between the distributions driven by the model and that of the experiment, i.e. fitting error (Marshall et al. 2016) which is indicated by KS_D_. The KS relation is

10 $$ {\text{KS}}(f,g) = {\text{Max}}\left| {{\text{CDF}}(f) - {\text{CDF}}(g)} \right|, $$

where CDF stands for the cumulative distribution function. By reproducing 1000 realizations to obtain $P<0.001$ with the model distribution, we can estimate the KS difference between the model and generated datasets, i.e. KS_G_. The KS between two typical distributions is illustrated in Fig. 2b.

The *P* value was defined as the number of reproduced ensembles we obtain ${\text{KS}}_{{\text{G}}}  \ge {\text{KS}}_{{\text{D}}}$ over all ensembles. If this *P* value has a large value, it implies that the fitting error is due to the random nature of the experiment, but if the *P* value is lower than 0.1, we can reject the model.

To evaluate the fitting parameters, low and high cutoffs must be specified. To keep the largest number of avalanches, we set the low cutoff $x_{{\min }}$ at 2 for size and duration distributions, and since the number of avalanches depends on the value of the threshold, which varies from one channel to another (see section for avalanche detection), we neglected rare avalanches that had a probability less than 0.01 when choosing the high cutoff. Therefore, $x_{{\max }}$ is the largest avalanche with $p(x)\ge 0.01$. The method is illustrated in Fig. 2c.

### Randomized controls of event cluster distributions

To investigate whether the observed power-law behavior was due to the methodology used, or could be attributed to the intrinsic correlation inherent in the data, we created randomized controls and repeated our analysis. For visual comparison, we plotted the avalanche size and duration of the original data and the randomized data in Fig. 7.

#### Method 1: randomizing the time intervals

As shown in Fig. 1a, first suprathreshold events were detected. Suppose some events with the occurrence times {4.6, 6.7, 9.8, 16.7, 22.5} which results in events’ intervals {2.1, 3.1, 6.9, 5.8}. Random values were assigned to each occurrence time. These random values were drawn from a uniform distribution in the range of zero and the length of the signal. New randomized occurrence times = {13.7, 6.2, 22.9, 18.6, 17.3}. Then the set was sorted by new occurrence times, and we will have the new intervals {7.5,3.6,1.3,4.3}. This approach preserved the number of events for each time series, yet, abolishes the corresponding inter-event intervals and thus correlations between time series.

#### Method 2: randomizing the original time series

The original data were shuffled entirely, i.e. we shuffled *y* values of the original EEG signal into different time points and suprathreshold events were detected from the new shuffled signal. This procedure changed both correlations and the number of events in the signal; therefore, the distribution of intervals and the number of events in the shuffled signal were different from the original signal.

### Detrended fluctuation analysis

The presence of neuronal avalanches is in line with critical state dynamics in which neuronal events exhibit long-range temporal correlations (LRTC) (Chialvo 2010). To study LRTCs between suprathreshold EEG events, we use detrended fluctuation analysis (DFA) (Kantelhardt et al. 2002) (see "Avalanche detection" for more details). We analyze the EEG suprathreshold events time series collected from all channels of all infants. In particular, events are collected at their occurrence times from all channels and for each subject to form a single time series (note that simultaneous events add up to a single large event, and then the interval between events are calculated).

To conduct the DFA analysis, first, the cumulative time series of given data *x* is calculated:

11 $$  Y(i) \equiv \sum\limits_{{k = 1}}^{i} {\left[ {x_{k}  - \bar{x}} \right]} ,\quad i = 1,2, \ldots N. $$

Then the series of non-overlapping segments of length *s* which we refer to as scale is driven from the cumulative signal. The signal is detrended and the variance of $\nu$th segment of length *s* is calculated as follows:

12 $$ F^{2} (s,\nu ) \equiv \frac{1}{s}\sum\limits_{{i = 1}}^{s} {\left\{ {Y\left[ {(\nu  - 1) \times s + i} \right] - y_{\nu } } \right\}^{2} } . $$

Average of the variances over segments gives the fluctuation function which can be calculated as follows:

13 $$ F(s) \equiv \left\{ {\frac{1}{{N_{s} }}\sum\limits_{{\nu  = 1}}^{{N_{s} }} {F^{2} } (s,\nu )} \right\}^{{1/2}} . $$

The fluctuation function can be fitted with a power function of scales as $F(s) \propto s^{h}$ and *h* is the Hurst exponent. A Hurst exponent $0.5< h <1$ demonstrates the existence of LRTC while $h = 0.5$ is corresponding to the uncorrelated signal (Parish et al. 2004; Benayoun et al. 2010).

The de-identified EEG data, documentation, and all code used in these analyses will be made available upon request to M. Z. and/or A. A. B.

### Statistical tests

We used two different statistical analyses throughout the study: (1) to select the best fit model for distributions, we calculated a *P* value for each model using KS test, based on procedures used in (Clauset et al. 2009). The details of the KS test are described in “Size and duration distributions of avalanches”. (2) To explore if there is a significant difference between avalanche distributions across age groups, we deployed the common Student’s *t* test on exponents corresponding to the distributions and report the *P* values; $P< 0.05$ indicates the significant difference.

## Results

**Fig. 3:**
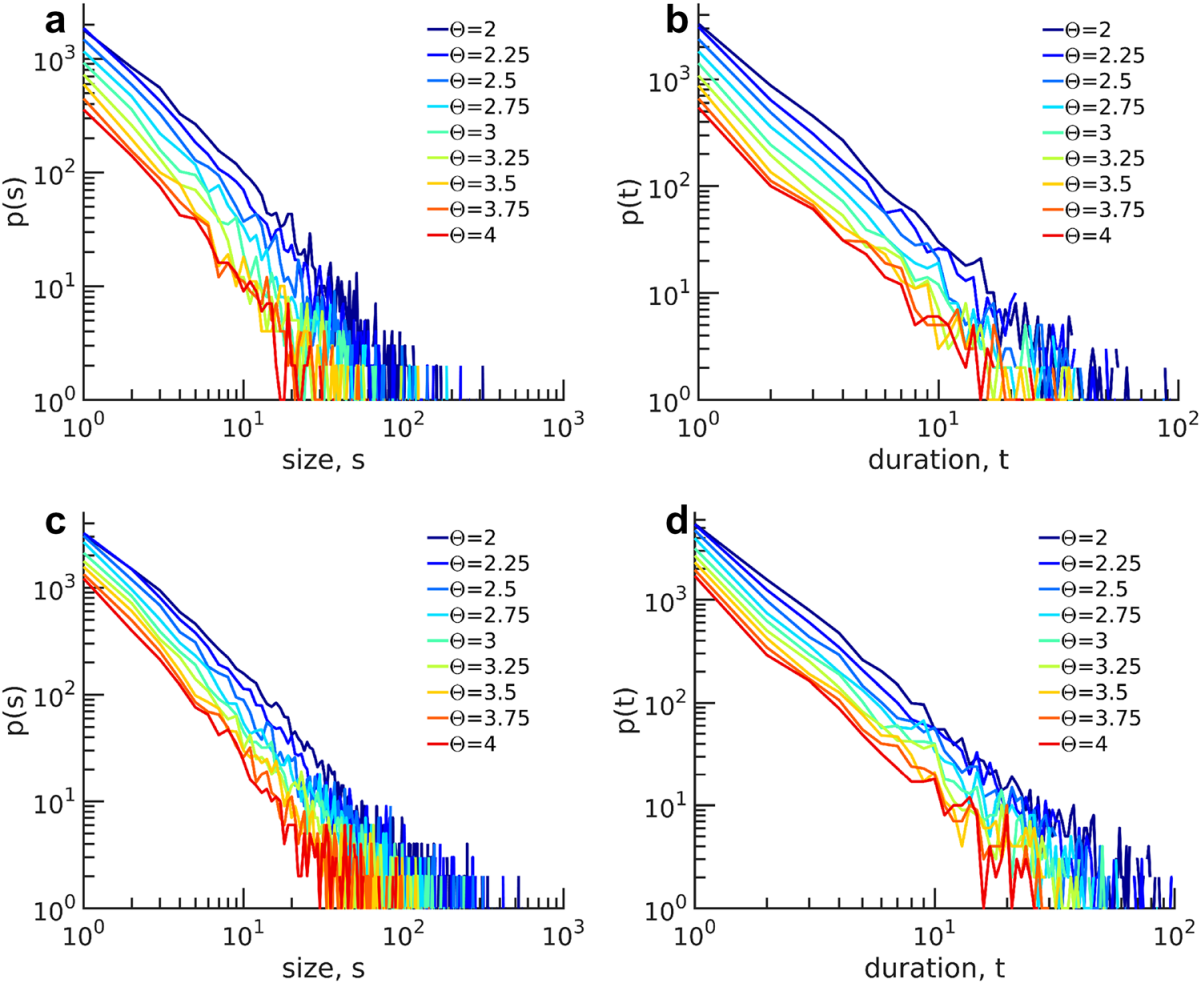
Avalanche distributions in size *s*, and duration *t* for subject #14 (**a**, **b**) at 6 months, (**c**, **d**) at 12 months. Different color lines refer to different values of the threshold. The distributions display a straight line for a range of *s* and *t* in double-logarithmic scales. The lines have the same slope for different thresholds, which demonstrates the robustness of power-law distributions against changes in the threshold

To identify scale-invariant features in the infant data, we applied avalanche analysis in size and duration for different thresholds ranging from 2 to 4 times the standard deviation (SD) for each channel in the full frequency band (i.e., over the entire uncategorized frequency band, i.e. 0.1–100 Hz). In Fig. 3a–d, we show the exemplary distributions of size and duration for subject #14 at 6 and 12 months of age respectively. Distributions display a straight line in a double-logarithmic scale, and remarkably, the slopes for different distributions are independent of the thresholds. The straight lines of the distributions in the double-logarithmic coordinate suggest a power-law organization of neuronal avalanches robust to threshold variation.

To find the best model for describing these distributions, we calculated the parameters using the maximum likelihood estimation (MLE) method (Clauset et al. 2009) for each model and rejected inappropriate models based on *P* values.

**Fig. 4:**
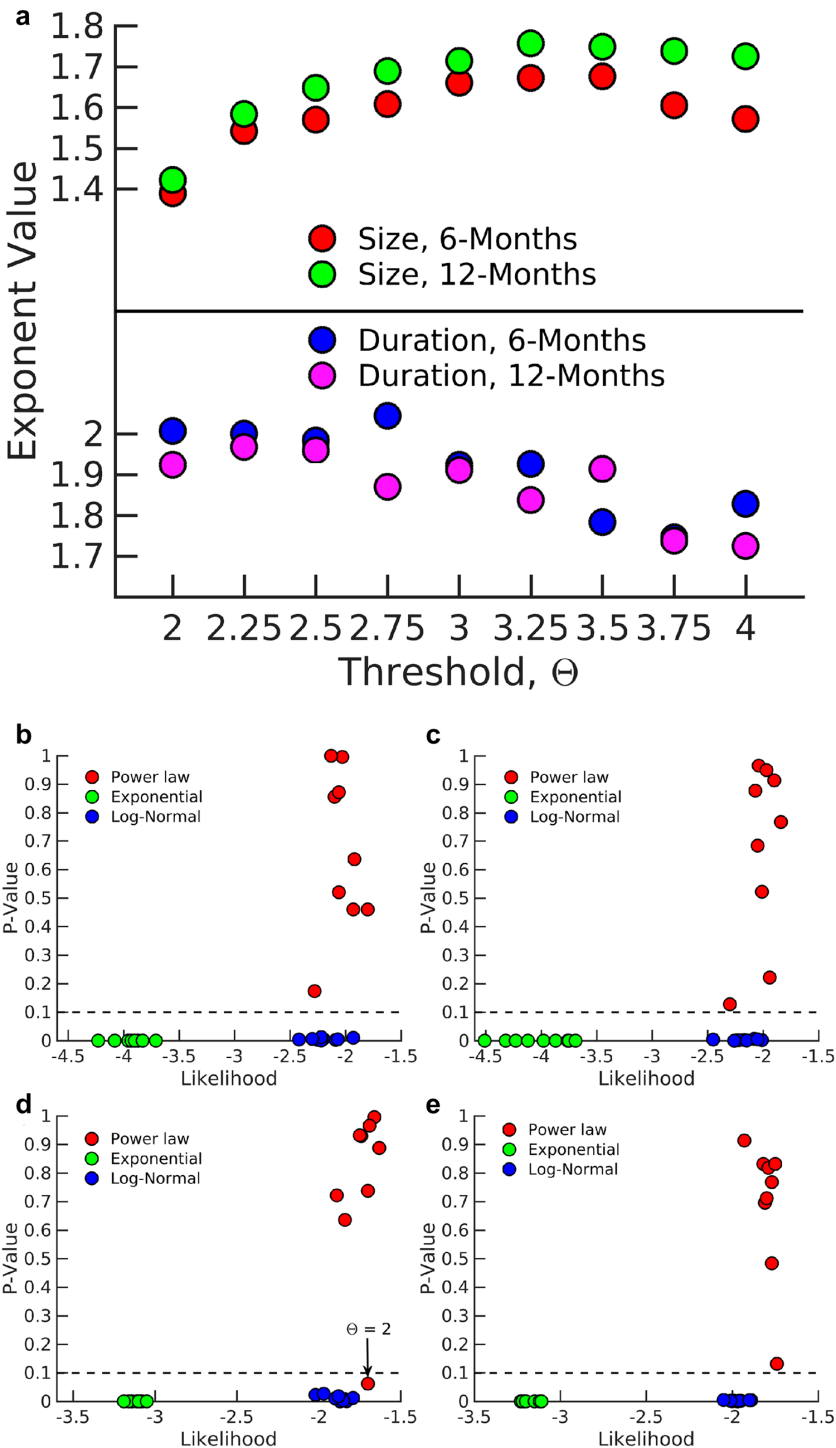
Model parameters for avalanche distributions. **a** The exponent of avalanche distribution in size and duration for subject #14 at 6 months and at 12 months for different thresholds. The exponents are robust against changing the threshold. *P* value and likelihood for different models for the distribution of **b**, **c** size and **d**, **e** duration of avalanches at 6 and 12 months, respectively. The *P* value for exponential and log-normal models are below 0.1 and these models are rejected. The power-law model has larger likelihood in most cases and is considered as the best fit model for distributions. Please note that the error bar for each parameter is too small to be plotted

*P* values of the power-law model in most cases were larger than 0.1, while the two exponential and log-normal models had very small *P* values. Specifically, the *P* value is zero for the exponential model, which indicates the minimum likelihood. Indeed, within the fitting range, the exponential model is not an appropriate model for the distributions since the tail of the distribution falls rapidly (see Fig. 2a in “Materials and methods”). Although the likelihood of the log-normal model is comparable to that of the power-law model, its *P* values were lower than the threshold and similar to the exponential model, we rejected log-normal models. We conclude that power-law is the model that best describes the empirically obtained avalanche distributions (see Fig. 4).

**Fig. 5:**
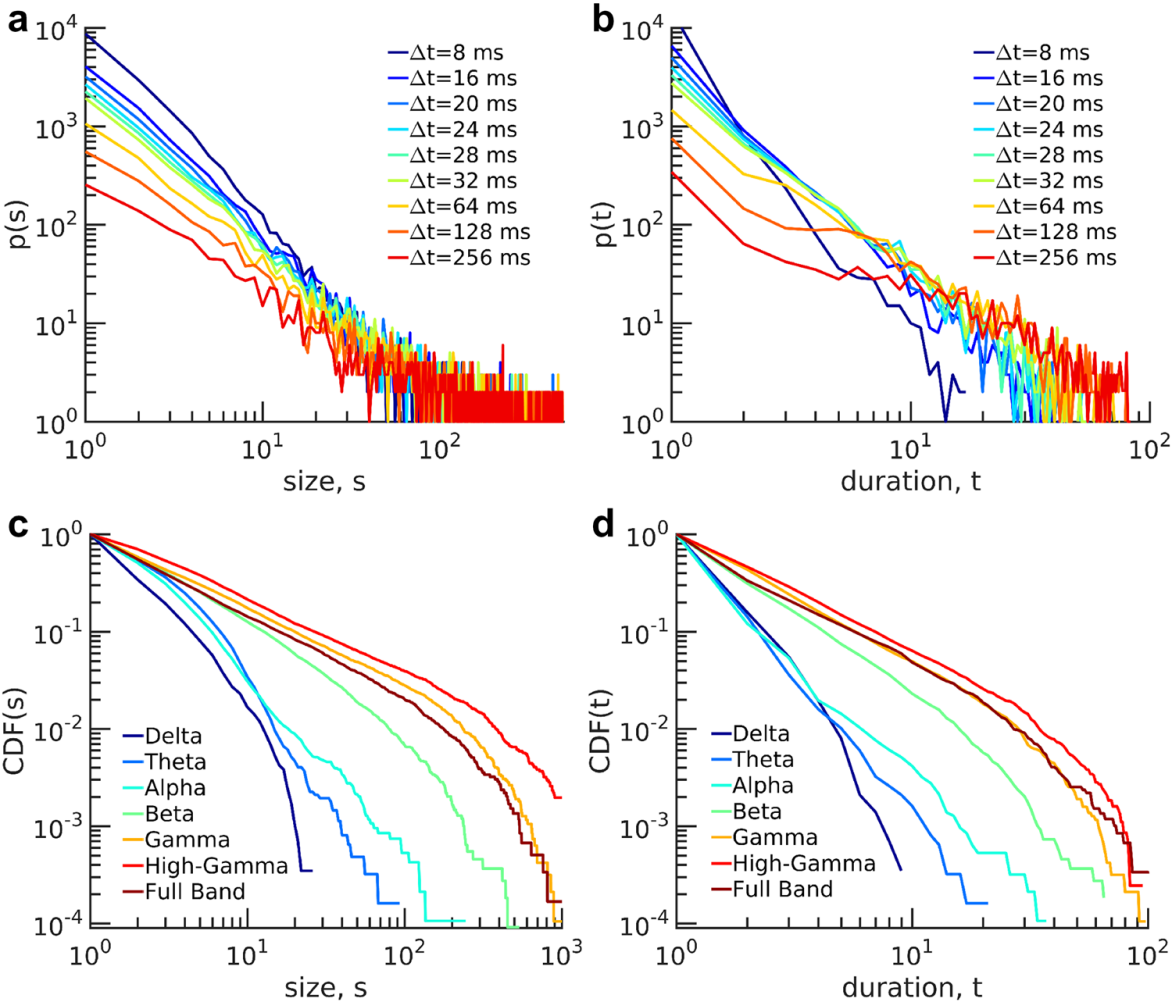
Avalanche distribution and variations of $\Delta t$ for subject #14 at 12 months. **a** Avalanche distribution in size and **b** avalanche duration for different separation times. Power-law behavior is independent of separation time in a specific range of $\Delta t$. CDF of **c** avalanche size, and **d** avalanche duration for different frequency bands. Power-law behavior is pronounced in high-frequency bands ($\beta$, $\gamma _{{\text{L}}}$, and $\gamma _{{\text{H}}}$), similar to full frequency band while distributions deviate from power-law in low-frequency bands ($\delta$, $\theta$, and $\alpha$)

In Fig. 5a, b, we plot avalanche size and duration distributions for different $\Delta t$s. We show that distributions in the range of $16 \le \Delta t \le 32$ ms are similar and conclude that the power-law behavior is independent of $\Delta t$ for this range. For our following analysis, we set $\Delta t= 24$ ms.

To evaluate a potential dependency of the power laws on the frequency content of the signal, we applied the avalanche analysis to different frequency bands. As shown in Fig. 5c, d, power-laws were found in the broad frequency band, $\beta$, $\gamma _{{\text{L}}}$, and $\gamma _{{\text{H}}}$ frequency bands. In contrast, avalanches calculated from low-frequency bands $\delta$, $\theta$, and $\alpha$ deviated from power-laws. Table 1 shows *P* values for the power-law model of avalanche distribution in size and duration for all frequency bands of subject #14 at 12 months.

**Fig. 6:**
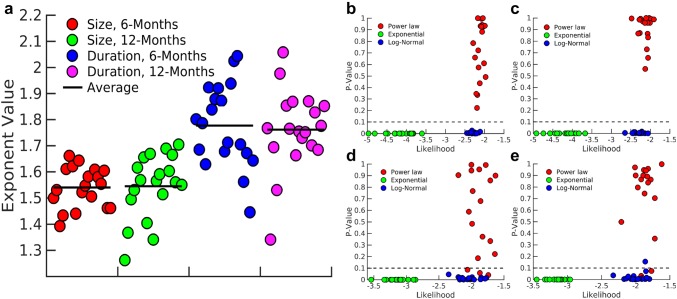
Model parameters for avalanche distributions for ALL subjects ($\varTheta =2.75$ SD, and $\Delta t= 24$ ms). **a** The exponents for avalanche size and duration distribution for all subjects at 6 and 12 months of age. The exponents show no significant difference at these two ages. *P* values and likelihood of each model for **b**, **c** size distribution and **d**, **e** duration distribution for 6- and 12-month-olds, respectively. The power-law model is the best-fitted model in all size distributions and for most of the duration distributions of avalanches

We evaluated the *P* values and likelihood ratio of avalanche size and duration distributions for all subjects at 6 and 12 months, respectively, for a chosen threshold $\varTheta =2.75$ SD and temporal resolution of $\Delta t = 24$ ms shown in Fig. 6. For most subjects, the avalanche size and duration were best described by a power-law distribution. The average exponent of the avalanche size distribution in 6-month-old infants was $\tau = 1.540 \pm 0.075$ and for 12-month-olds was $1.545 \pm 0.121$. These values were very close to the mean-field exponent of size distribution, i.e. $\tau =1.5$ (Sethna et al. 2001), whereas the average of duration distribution exponents, $\alpha$ is $1.777 \pm 0.156$ for 6-month-olds, and $1.761 \pm 0.151$ for 12-month-olds which differed from the mean-field value $\alpha = 2$ (Sethna et al. 2001). Paired *t* tests revealed no significant difference across ages ($P = 0.886$ for avalanche size, and $P = 0.762$ for duration distributions).

**Table 1:** *P* values for the power-law model in avalanche distribution in size and duration for subject #14 at 12 months

Frequency band	*P* value for power-law model	
	Size	Duration
$\delta$	0.04	0.80
$\theta$	0.00	0.04
$\alpha$	0.00	0.00
$\beta$	0.16	0.25
$\gamma _{{\text{L}}}$	0.75	0.96
$\gamma _{{\text{H}}}$	0.18	0.15

The avalanche distributions deviate from power-law behavior in low-frequency bands

**Fig. 7:**
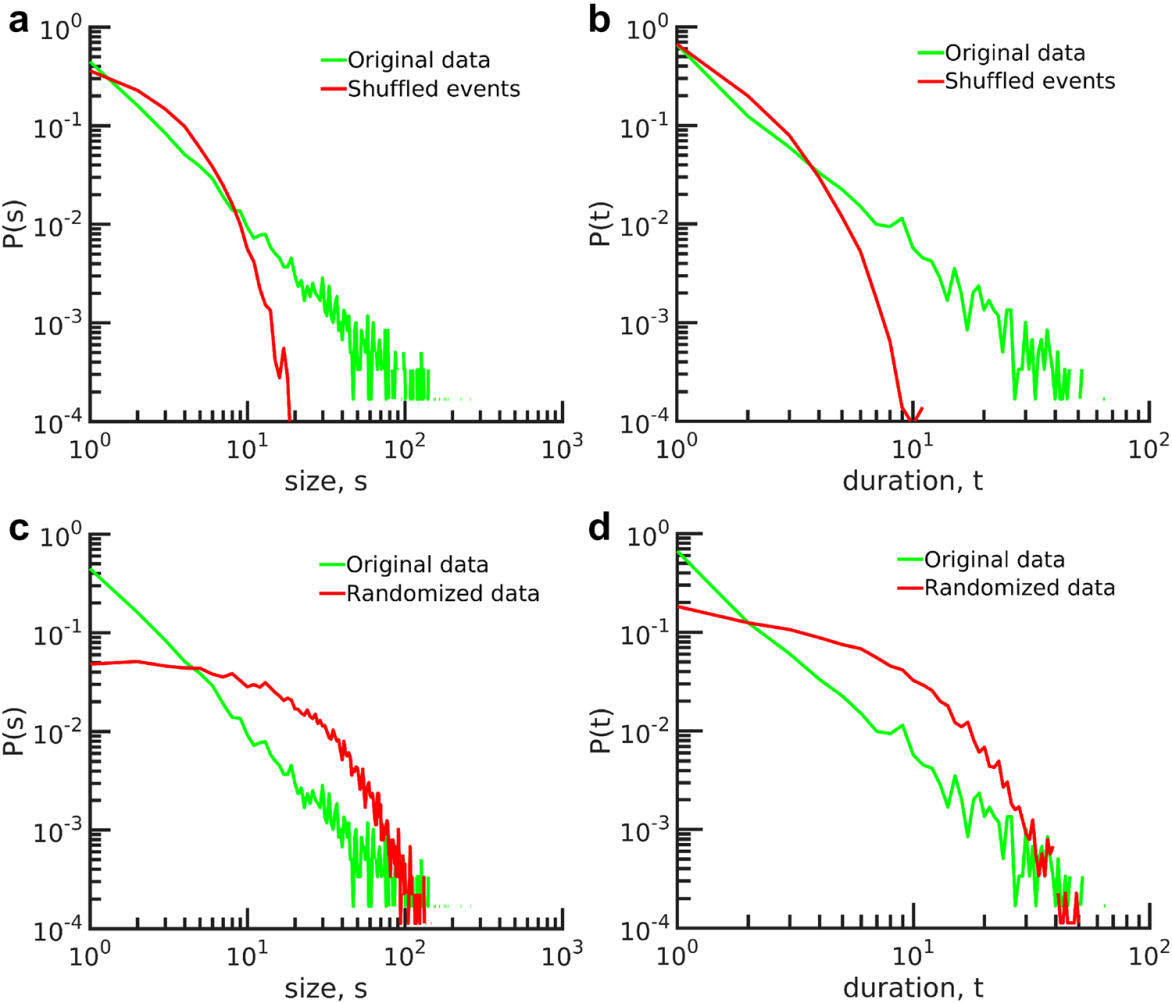
Distribution of randomized controls of event cluster. Avalanche distribution in size and duration by **a**, **b** randomizing the time intervals, **c**, **d** randomizing the original time series. The distributions of avalanche size and duration in randomized time intervals show that power-law behavior is associated with correlations in data. The distributions of avalanche size and duration in randomized data show that power-law behavior is irrelevant to the number of activities. All plots represent data from subject #14 at 12 months and $\varTheta =2.75$ SD

Analysis of shuffled controls is presented in Fig. 7 and Table 2. The distributions of event clusters obtained from shuffled data displayed an exponential function and significantly deviated from a power-law distribution. We conclude that the emergence of power-law behavior in the infant EEG arises from the correlations across EEG electrode sites and that those correlations were destroyed by shuffling. Table 2 provides quantitative details on *P* value analysis of distributions in size and duration.

**Table 2:** *P* value analysis of distributions in size and duration, original signal compared to shuffled and randomized data

	Size distribution			Duration distribution		
	Power-law	Exponential	Log-normal	Power-law	Exponential	Log-normal
Original signal	0.94	0.00	0.00	0.61	0.00	0.03
Shuffled events	0.00	0.00	0.02	0.08	0.00	0.41
Randomized data	0.00	0.95	0.02	0.00	0.98	0.04

**Fig. 8:**
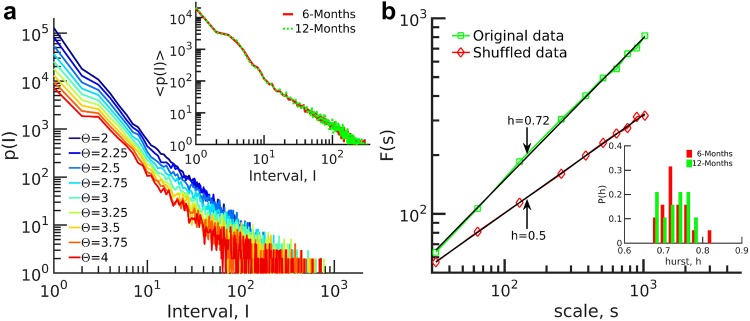
Distribution of peak intervals for subject #14 at 12 months at $\varTheta =2.75$ SD. **a** The distributions display a straight line on a bi-logarithmic scale which is similar for different thresholds. Inset: Average distribution over subjects for all the 6-month-old and 12-month-old infants. The distributions exhibit a scale-invariant behavior in intervals. **b** Fluctuations over scales and the Hurst exponent of peak intervals. The Hurst exponent of original data is $h > 0.5$ and demonstrates LRTC which is destroyed by shuffling the data. The distribution of *h* for all subjects at two ages is shown in the inset of **b**. No significant difference was detected between the two ages

To extract the statistical features of peak intervals, we calculated the distribution of peak intervals for different thresholds as depicted in Fig. 8a. The distributions exhibit a straight line in bi-logarithmic coordinate, resembling each other for different thresholds. These distributions, in agreement with previous studies, represent power-law behavior in the peak interval of activities (Zare and Grigolini 2012, 2013). The average distribution over all subjects is shown in the inset of Fig. 8a. The average distributions reflected very similar behavior and there was no significant difference between the two age points.

Neuronal avalanches are suggestive of critical dynamics which support the emergence of long-range temporal correlations (LRTC) in complex systems. Accordingly, we directly estimated LRTCs using detrended fluctuation analysis (DFA) (Kantelhardt et al. 2002; Kello et al. 2010; Palva et al. 2013), and analyzed the interval of EEG suprathreshold events time series collected from all channels of each infant. Events are collected at their occurrence times from all channels and for each subject to form a single time series. Note that simultaneous events add up to a single large event, and then the interval between events are calculated. We then calculated the Hurst exponent (Linkenkaer-Hansen et al. 2001). A Hurst exponent $h>0.5$ suggests the existence of LRTCs. As an example, the results for subject #14 at 12 months are shown in Fig. 8b. The Hurst exponent for the original time series was $h = 0.723$ demonstrating the existence of LRTCs. As a control, we destroyed temporal correlations by shuffling time series of suprathreshold events, which revealed $h = 0.5$ in line with expectation for uncorrelated activity.

The distribution of the Hurst exponent for all subjects is illustrated in the inset of Fig. 8b. The averaged Hurst exponent for 6-month-old infants was $0.728 \pm 0.035$ and it was $0.729 \pm 0.036$ for 12-month-old infants. The results of interval analysis did not reveal any significant differences across age (the *P* value of *t* test for Hurst exponents was $P = 0.996$) coinciding with the avalanche analysis discussed above.

## Discussion

Several studies have shown trajectories of early human brain growth and have probed the association between brain activity and brain growth in preterm and neonatal infants (Kostović and Judaš 2006; Hüppi et al. 1998; Hüppi and Dubois 2006; Kostović and Judaš 2008; Benders et al. 2015). However, the main challenge for the developing infant brain, as neuronal connections rapidly proliferate and mature, is the maintenance of stable neuronal dynamics that allows for reliable information processing. Here we report the hallmark of neural avalanches evident across the first year of life; specifically, we demonstrate that suprathreshold events from dense array scalp EEG organize as spatiotemporal clusters whose distributions in size and duration follow power-laws. No age differences in these events were detected between 6 and 12 months of age, suggesting that avalanche dynamics are already well established by 6 months and are maintained throughout the first year of life. Our findings are in line with studies in adult humans that examined ongoing activity using fMRI (Tagliazucchi et al. 2012) and the local field potential in adult nonhuman primates (Petermann et al. 2009). Infant avalanches demonstrated threshold independence in their scale invariance, that is, large and small local amplitude events in the event are similarly organized. Such an *avalanche within an avalanche* structure indicates a specific organization in the amplitude of local suprathreshold EEG events as found for LFP avalanches in nonhuman primates and in the ECoG and fMRI of ongoing activity in humans (Petermann et al. 2009; Tagliazucchi et al. 2012). Assuming that the amplitude of local events in the EEG correlates with neuronal group synchronization, this organization complements scale invariance in space and time. In the present study, we demonstrate this for the first time in typically developing infants during sensory processing across the first year of life.

Previously, scale-free neural activity was reported in pre-term and full-term newborn infants. But as specific EEG patterns occur across different stages of development, it is unknown whether the bursting, scale-free activity seen in preterm infants is the same as the scale-free avalanche activity seen outside the newborn period. The premature infant’s EEG (from 24-week gestational age) evolves from an “asymmetric discontinuous” activity to the “symmetrically continuous” pattern seen in full term infants (between 40- and 44-week gestational age). Intermittent bursts of electrical activity predominantly seen in the delta range (0.1–0.5Hz) in the preterm period, are termed spontaneous activity transit (SAT), and have been suggested to constitute a physiological entity per se (Vanhatalo and Kaila 2006). Although it has been demonstrated that SATs exhibit scale-free properties (Iyer et al. 2015), characteristically, SATs are no longer seen after the “term” age period and when seen after that period, they are associated with pathological conditions including asphyxia at birth (Iyer et al. 2014; Roberts et al. 2014). Although Roberts et al. (2014) “position the neuronal activity in neonatal HIE (hypoxic–ischemic encephalopathy) within a broad class of irregular physical processes, known as avalanches”, the authors are still discussing HIE. Due to the fact that “burst suppression” is considered an abnormal, asymmetric pattern (Niedermeyer et al. 1999) usually associated with hypoxia, and SATs are a transitory phenomenon, this neural activity does not seem to be categorically the same in nature as the avalanche activity reported here, found well beyond the neonatal period in typical infant development. To our knowledge, evidence of the presence of neuronal avalanches in typical infant development or about the timing in which avalanche dynamics is stable has not yet been reported. Establishing normative avalanche behavior is thus critical to identifying deviations during the course of atypical development or clinical conditions.

Two of the novel methodologies applied in this study include the use of multi-site recordings and inspection of broadband frequency content. Dense-array recordings allowed examination of avalanche formation at both local sites and as a collective signature of the global system. On the other hand, information obtained only by local recording focusing on bursts provides a one-dimensional statistical measure restricting correlational analysis. We identified suprathreshold events from all 62 channels, while in previous work (Iyers et al. 2014, 2015, Roberts et al. 2014), a biparietal channel (P3–P4) montage localized activity in reduced space-time, since bursts captured from an EEG channel represent neural activities in a small area ($\sim 5$ cm) with a maximum duration of around 5 s. Neuronal avalanches are a more global feature of brain dynamics which spread activity throughout the brain. Thus, avalanches can more easily be correlated with high-level brain tasks, for example, the processing of complexity (Timme et al. 2016) used for consciousness quantification (Tononi and Edelman 1998). In this study, assuming that the amplitude of local events in the dense-array EEG correlates with neuronal group synchronization, we demonstrate for the first time that over the course of 6 months of development, distributions in size and duration of spatiotemporal clusters of activities from multi-site recording display power-laws. Our broadband spectral analysis revealed robust scale invariant cluster sizes that were dependent on the specific frequency band analyzed, that is, local events that arose from gamma-activity exhibited scale-invariant clusters, whereas scale invariance decreased when analyses were restricted to lower frequencies. These results suggest that the nesting of physiological frequencies is compatible with avalanche dynamics as reported previously in post-natal rodents (Gireesh and Plenz 2008).

It has recently been suggested that certain uncorrelated local event activity, due to spatial neighborhood overlap, can give rise to scale-invariant cluster size distributions (Touboul and Destexhe 2017). These conditions were not met in our infant EEG data sets as (1) randomizing destroyed power law statistics and (2) local events were significantly correlated over time. In contrast, scale-free fluctuations in the spatial extent of neuronal activity in conjunction with long-range temporal correlations (LRTC) are considered to be a signature of criticality (Chialvo 2010). Theory and experiments have shown that networks that are at or near criticality exhibit efficient information processing (Beggs and Plenz 2003; Socolar and Kauffman 2003; Shew et al. 2009; Beggs and Timme 2012; Friedman et al. 2012; Yang et al. 2012), including maximal information storage and capacity (Socolar and Kauffman 2003; Shew et al. 2009), maximal dynamic range (Kinouchi and Copelli 2006; Shew et al. 2011) and information transition (Beggs and Plenz 2003), optimal communication (Beggs and Plenz 2003; Bertschinger and Natschäger 2004; Rämö et al. 2007; Tanaka et al. 2009), high computational power (Bertschinger and Natschäger 2004) and maximal variability in phase synchrony (Yang et al. 2012). Accordingly, criticality appears to be signature of a healthy brain (Massobrio et al. 2015) that is able to flexibly adapt to rapidly changing environments (Chialvo 2010). This ability to process efficiently and flexibly adapt to rapidly changing environments is even more critical to the developing brain.

However, it is the case that power-laws are suggestive of criticality, rather than proof of it. Hence, in this study, we use a series of analyses to investigate whether the observed power-law behavior was due to the methodology used or could be attributed to the intrinsic correlation inherent in the data. LRTCs between suprathreshold EEG events, examined by Detrended Fluctuation Analysis (DFA), clearly suggested that the emergence of power-law behavior in the infant EEG arises from the correlations across EEG electrode sites, correlations that were destroyed by shuffling. Calculation of the Hurst exponent for the original time series demonstrated the existence of LRTCs, whereas in the shuffled data, time series of suprathreshold events were found in line with expectation for uncorrelated activity.

Nevertheless, there is no consensus on the critical brain hypothesis among the scientific community. A group of critics believe that power laws are ubiquitous in nature, something that might not be particular to brain function and can potentially emerge from noise (Touboul and Destexhe 2017). A couple of similar studies have discussed the proper power-law model and functional fit (Clauset et al. 2009; Dehghani et al. 2012) emphasizing the careful identification of power law cut-offs in avalanche distributions, and the incorporation of them into appropriate statistical models (Yu et al. 2014). These critiques have refocused the debate on the specific, shallow exponents found for avalanche power-laws. Alternative approaches to avalanche dynamics using temporal scaling (Hardstone et al. 2012) and spatial scaling of fluctuations in ongoing human brain activity (Haimovici et al. 2013) have brought further support to the hypothesis of criticality in the brain.

It could be the case that this neural activity is just a ubiquitous property of brain networks and unfortunately, we do not have a clinical index in this manuscript. To clarify whether power-law behavior is a ubiquitous property of brain networks, there is still work to be done using comparable methodology and technical details, from data acquisition to in-depth analysis. For instance, research on scaling laws has presented distinct arguments and evidence. We do agree that any fundamental approach to scaling laws in cognitive science will need to explain the variability observed in scaling law parameters estimated from data (Kello et al. 2010). Scaling laws are measured by exponents, and exponents vary across individuals (Holden et al. 2009; Gilden and Hancock 2007), across tasks (Kello et al. 2007) and across time (Ihlen and Vereijken 2010). It is still an open question how variability in scaling laws reflects the relevant adversities. There are indeed numerous studies that claim that scale-free behavior is a ubiquitous property related to both the dynamics and structure of brain networks (Eguiluz et al. 2005; Bullmore and Sporns 2009; Chialvo 2004; He et al. 2010). But, if power-law is indeed a ubiquitous property in biological systems including the brain networks, it would still be a potential biomarker for the system itself when it deviates from the processes or patterns that are repeated across scales of analysis.

Considering our results and the insight the cited studies offer, we propose that the stability of neuronal avalanches denotes healthy brain development across infancy and, therefore, has the potential of being a valuable biomarker. Further, that deviation in avalanche structure and stability and thus criticality may indicate a disordered trajectory of maturation and consequently serve as a reliable prognostic measure of not just concurrent, but also more permanent disabilities (Iyer et al. 2014; Massobrio et al. 2015). Therefore, our findings support the development of early biomarkers within the framework of criticality that have the potential to aid diagnosis and treatment of pathological states in human infants.

## Conclusion

We found robust evidence of the stability of neuronal avalanches in early infancy. This result suggests that long-range temporal correlation already exists over the first year of life during the early stages of ongoing brain maturation. Previously, the presence of scale-free dynamics in specific brain areas in neonates was reported in preterm infants and full-term asphyxiated newborns (Roberts et al. 2014; Iyer et al. 2015). Here, using dense-array EEG recordings, the emergence of power-law behavior in large-scale dynamics in infants during auditory sensory processing was identified. The neuronal avalanche mechanism was invariant across two age points, due to the intrinsic global nature of neural avalanches. Our results suggest that the propagation of neocortical activities is scale invariant during infancy, which may help the brain to maintain stable neuronal dynamics while allowing continued optimized conditions for critical information processing. The fact that we did not find across age differences in power-law behavior supports the hypothesis that power-law behavior, or specifically “scale-invariance of neural dynamics” is an inherent feature of the infant brain that is already evident across the first year. Moreover, these results support our premise that the infant’s brain self-organizes to a stable critical state that will allow for optimal information processing across this period of rapid cognitive development. Importantly, demonstration of neural avalanches in the infant’s brain constitutes a critical first step towards using dynamical biomarkers to predict current biological risk or identify early signs of developmental disorders.
